# First-Principles Study of Ir_n_ (n = 3–5) Clusters Adsorbed on Graphene and Hexagonal Boron Nitride: Structural and Magnetic Properties

**DOI:** 10.3390/nano12142436

**Published:** 2022-07-16

**Authors:** Mei Ge, Leiting Chu, Miaomiao Guo, Yan Su, Junfeng Zhang

**Affiliations:** 1 School of Physics and Information Engineering, Shanxi Normal University, Taiyuan 030031, China; gemei@sxnu.edu.cn (M.G.); chult123@163.com (L.C.); guomm95@163.com (M.G.); 2 Key Laboratory of Spectral Measurement and Analysis of Shanxi Province, Shanxi Normal University, Taiyuan 030031, China; 3 Key Laboratory of Materials Modification by Laser, Electron, and Ion Beams, Dalian University of Technology, Ministry of Education, Dalian 116024, China; su.yan@dlut.edu.cn

**Keywords:** clusters, graphene, hexagonal boron nitride, substrate effect, magnetic property

## Abstract

Magnetic clusters have attracted great attention and interest due to their novel electronic properties, and they have potential applications in nanoscale information storage devices and spintronics. The interaction between magnetic clusters and substrates is still one of the challenging research focuses. Here, by using the density functional theory (DFT), we study the structural stability and magnetic properties of iridium clusters (Ir_n_, n = 3–5) adsorbed on two-dimensional (2D) substrates, such as graphene and hexagonal boron nitride (*h*BN). We find that the most favorable configurations of free Ir_n_ clusters change when adsorbed on 2D substrates. In the meantime, the magnetic moments of the most stable Ir_n_ reduce to 53% (graphene) and 23.6% (*h*BN) compared with those of the free−standing ones. Interestingly, about 12-times enlargement on the magnetic anisotropy energy can be found on *h*BN substrates. These theoretical results indicate that the cluster–substrate interaction has vital effects on the properties of Ir_n_ clusters.

## 1. Introduction

Owing to unexpected electronic and magnetic properties, atomic clusters are promising candidates for technological applications such as catalysis and magnetic storage. They have been becoming gradually more and more attractive in interdisciplinary fields since the 1990s [1,2,3]. The size and atomic structure-dependent properties of clusters present a huge opportunity for designing cluster-assembled materials and devices. Therefore, determining the ground state structure of a cluster directly by combining experimental and theoretical techniques is vital [1,4].

Magnetic clusters have attracted special attention, especially those composed of transition metals (TM) [5]. Because of the strong spin–orbital coupling (SOC), it is expected that nanostructures containing TM atoms will possess possibly greater magnetic anisotropy energy (MAE), which is a key parameter in nano-information storage devices. Generally, with the increase in the atom number *n*, the geometric structure of clusters will undergo a transition from line models (n = 2–3) to planar models (n = 3–5) and then to three-dimensional configurations (such as a pyramid, octahedron, icosahedron, cube or core-shell model) [6,7,8,9,10,11,12,13]. For instance, owing to the *s(p)-d* hybrid, there is a predominantly icosahedral growth for Fe_13_ and Pd_13_ clusters [14,15]. The electronic and magnetic properties of magnetic clusters highly depend on the atomic size. As the cluster size increases, an odd–even oscillation of vertical ionization potentials occurs in Ir_n_ (n < 8) and Rh_n_ (n < 13) clusters [16,17]. The strongly quenched orbital magnetic moment in size-dependent Fe_n_ (n = 3-20) clusters is obtained experimentally [14] and then confirmed by DFT calculations [18]. Furthermore, the size effects on magnetic moments have also been observed in Ni_n_ (n = 10–15) [19,20], Rh_n_ (n = 2–13) [3,16], Pd_n_ (n = 2–23, 55, 147) [15], Pt_n_ (n = 2–20) [21] and Ir_n_ (n = 1–13) clusters [22].

Generally, clusters can be experimentally prepared by several methods, such as magnetron sputtering [23], laser vaporization [24,25,26] and chemical vapor deposition (CVD) [27]. Normally, a substrate is necessary for many applications of the clusters. The interaction between the cluster and substrate can affect both the geometric structure and physical properties of a cluster. For instance, under the influence of the cluster–substrate interaction, Fe_n_ (n = 2–7), (Mn_n_ (n = 2–7) and Si_n_ (n = 2–6, 10)) clusters on graphene prefer different growth modes and various orientations [28,29]. Additionally, the electronic and magnetic properties of the substrate can be effectively modulated by the adsorption of clusters [30,31,32,33]. When the Mn_5_ cluster is absorbed on graphene, the magnetic moment of the Mn_5_ cluster is enhanced by 186% because of the electron redistribution [29]. On the contrary, for Fe_n_ (n = 1, 4–6) clusters, the magnetic moment is reduced by 2–4 μ_B_ [28]. Furthermore, magnetic clusters can turn into stable nonmagnetic clusters when adsorbed on 2D substrates [33]. Interestingly, a large MAE can be achieved by absorbing clusters on substrates. Among them, Ir_n_ is a suitable candidate for nano-information storage devices and has been investigated extensively. Hu et al. [34] put an Ir_2_ dimer on the double vacancy site of 2D hexagonal boron nitride (*h*BN) and obtained an enlargement in MAE (~126 meV) [35]. Meanwhile, we have also investigated the substrate effect on MAE of Ir_2_ and found that MAE depends on the adsorption site and density [36]. However, from our limited knowledge, the interactions between magnetic clusters and substrates are still far from well known, especially on the MAE of larger clusters.

In this paper, by using first-principles calculations, we systematically study the interactions between Ir_n_ (n = 3–5) clusters and 2D substrates (graphene, *h*BN and germanene) [37]. Different ground state geometric structures of Ir_n_ and absorption sites of substrates are considered. We first investigate the geometric structures and stabilities with the help of cohesive and detachment energies. Then, we discuss the magnetic properties, including the magnetic moments and MAE, and explore the physics picture with the help of the local density of states and perturbation theory analysis. The present theoretical studies will provide insight into the substrate effect on larger magnetic clusters.

## 2. Materials and Methods

The structural, magnetic and electronic properties of Ir_n_ (n = 3–5) clusters adsorbed on 2D materials were studied by first-principles calculations, as implemented in the Vienna Ab-initio Simulation Package (VASP) code [38]. The ion–electron interaction was treated with the projector-augmented plane wave (PAW) potentials [39], and the exchange-correlation potential was described by generalized gradient approximation (GGA) with the Perdew–Burke–Ernzerhof (PBE) functional [40]. The wave functions were expanded in a plane wave basis set with an energy cut-off of 500 eV. The 2D substrates were chosen as 7 × 7 graphene, 7 × 7 *h*BN and 4 × 4 germanene supercells, respectively. To avoid the interaction between two neighboring layers, a vacuum space of 40 Å was added along the Z-direction. A *k*-mesh of 3 × 3 × 1 was used for the Brillouin zone. The atomic structures were fully relaxed without any symmetric constraints, with total energy and force convergence criteria of 10^−4^ eV and 0.01 eV/Å, respectively. For the calculations of the magnetic and electronic properties, the convergence criterion for the total energy was set to 10^−6^ eV, and the SOC effects were considered.

We employed binding energy (*E*_b_, eV), cohesive energy (*E*_coh_, eV/atom) and detachment energy (*E*_det_, eV) to determine the most energetically favorable configuration of Ir_n_/2D. *E*_b_ can be used to evaluate the interaction between the Ir_n_ cluster and the 2D substrate, which is defined as:

*E*_b_ = *E*_Ir_n__ + *E*_2D_ − *E*_Ir_n_/2D_ (1)

where *E*_Ir_n__, *E*_2D_ and *E*_Ir_n_/2D_ are the total energies of the free−standing Ir_n_ cluster, 2D substrate (graphene or *h*BN) and Ir_n_/2D hybrid system, respectively. A larger *E*_b_ means a stronger interaction between Ir_n_ and the 2D materials. *E*_coh_ is the energy gain when isolated Ir atoms are assembled into the Ir_n_ cluster, which is defined as:

*E*_coh_ = (n × *E*_Ir_ − *E*_Ir_n__)/n or *E*_coh_ = (*E*_2D_ + n × *E*_Ir_ − *E*_Ir_n_/2D_)/n (2)

Here, n is the number of Ir atoms of the Ir_n_ cluster, *E*_Ir_ is the energy of an isolated Ir atom, *E*_Ir_n__ is the total energy of the free−standing Ir_n_ cluster and *E*_2D_ is the total energy of the 2D substrate, respectively. *E*_det_ is the energy gain when the Ir_n−1_ cluster is transformed to Ir_n_ by adding one more Ir atom, which can be used to determine the most favorable cluster size n. For the free−standing cluster Ir_n_ or that on the 2D substrate, the detachment energy can be defined as:

*E*_det_ = *E*_Ir_n−1__ + *E*_Ir_ − *E*_Ir_n__ or *E*_det_ = *E*_Ir_n−1_/2D_ + *E*_Ir/2D_ − *E*_Ir_n_/2D_ − *E*_2D_ (3)

where *E*_Ir_n−1_/2D_ and *E*_Ir/2D_ are the total energies of the Ir_n−1_/2D and Ir/2D hybrid systems, respectively.

## 3. Results

### 3.1. Structural Properties

**Figure 1:**
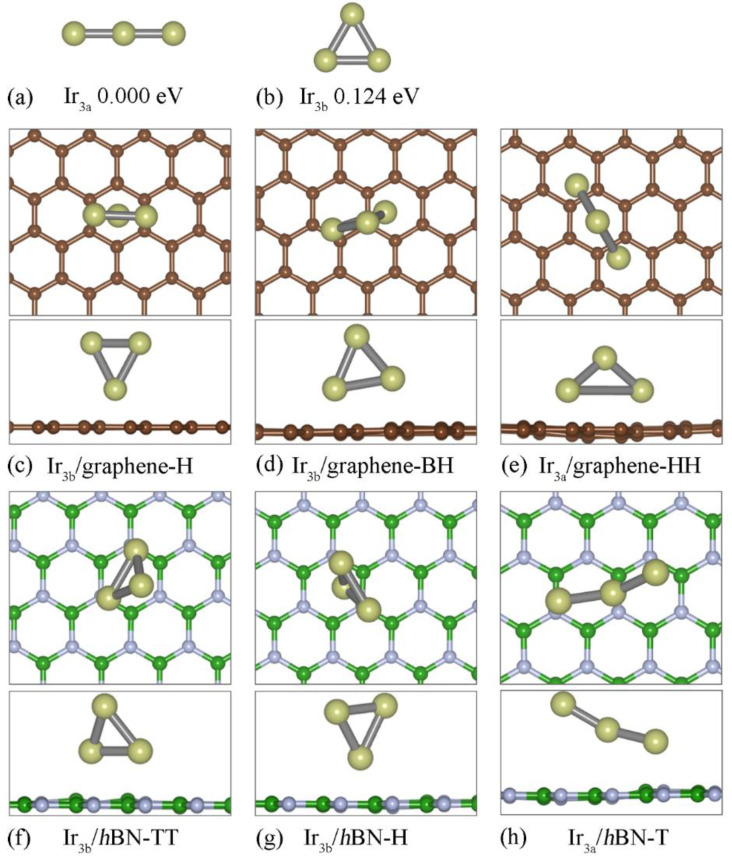
Atomic structures of the (**a**) Ir_3a_ (line model) and (**b**) Ir_3b_ (triangle model) clusters. Top and side view of the three relative stable atomic structures of the Ir_3_ cluster adsorbed on the graphene (**c**–**e**) and *h*BN (**f**–**h**) substrate, respectively. The Ir, C, B and N atoms are labeled with golden, brown, green and gray balls, respectively.

We investigated the structural, electronic and magnetic properties of the magnetic Ir_n_ clusters on different 2D substrates and then compared them with the free−standing ones. For the Ir_n_ (n = 3, 4, 5) clusters, we considered different isomers corresponding to the most stable configuration and the metastable ones. We then put the Ir_n_ cluster above three kinds of substates: graphene, *h*BN and germanene, respectively. Owing to the stronger interaction between the Ir and Ge atoms, the Ir_n_ cluster will dissociate and embed into the monolayer germanene, which can induce defected germanene. Taking Ir_3_ clusters as the examples, we show the optimized configurations of Ir_3_ adsorbed on germanene (see Supplementary Figure S1). Therefore, differently from our previous work in which the favorite adsorption site for the Ir_2_ cluster was the single vacancy of germanene [36], we mainly discussed the graphene and *h*BN substrates. Three kinds of absorption sites have been considered: on the top of an atom (T), on the top of a C-C (B-N) bond (B) and on the top of a hexagonal-ring-center (H). Accordingly, the Ir_n_ cluster absorbed on the 2D substrate is named Ir_nm_/2D-T (or B, H; see Figure 1), where n = 3, 4, 5 is the number of Ir atoms, m = a, b, c labels different isomers of free−standing Ir_n_ clusters, 2D is either graphene or *h*BN and T (or B, H) labels the absorption site of the nearest Ir atom.

As shown in Figure 1a,b, there are two relative stable configurations for the free−standing Ir_3_ cluster: the line model (Ir_3a_) and the triangle model (Ir_3b_). Hereafter, we set the energy of the most stable configuration as zero and ΔE as the energy difference between the most stable and metastable configuration, as listed in Table 1. Ir_3a_ is more stable than Ir_3b_, with an energy difference of 0.124 eV. Ir_3a_ has an average bond length of 2.181 Å. These results are consistent with most previous PBE calculations [22,41] and PW91 calculations [33]. However, when absorbed on graphene or the *h*BN substrate, the Ir_3_ cluster prefers the triangle model because of the substrate effect (see Figure 1c–h), which is similar to the Si_3_ cluster absorbed on the graphene substrate [42]. ΔE is 0.159 eV (on graphene) and 0.268 eV (on *h*BN), respectively. For the graphene substrate, the most stable configuration is the Ir_3b_/graphene-H (Figure 1c) hybrid system, in which the Ir_3_ plane is perpendicular to the graphene sheet, the innermost Ir atom is located at the H site with a *d* (distance between the Ir atom and the substrate) of 1.771 Å and the other two Ir atoms are located at the B site. As opposed to Ir_3b_/graphene-H, the most stable configuration is Ir_3b_/*h*BN-TT (Figure 1f), in which the plane of the Ir_3_ cluster has a tilt angle of 76.5° with a horizontal *h*BN sheet. Compared with Ir_3b_/graphene-H, there is an inversion for the triangle model, in which two nearer absorbed Ir atoms form chemical bonds with N atoms with a *d* of 2.248 Å. Owing to two Ir-N bonds, Ir_3b_/*h*BN-TT has an *E*_b_ of 5.583 eV, which is higher than that of Ir_3a_/graphene-H (5.352 eV).

**Figure 2:**
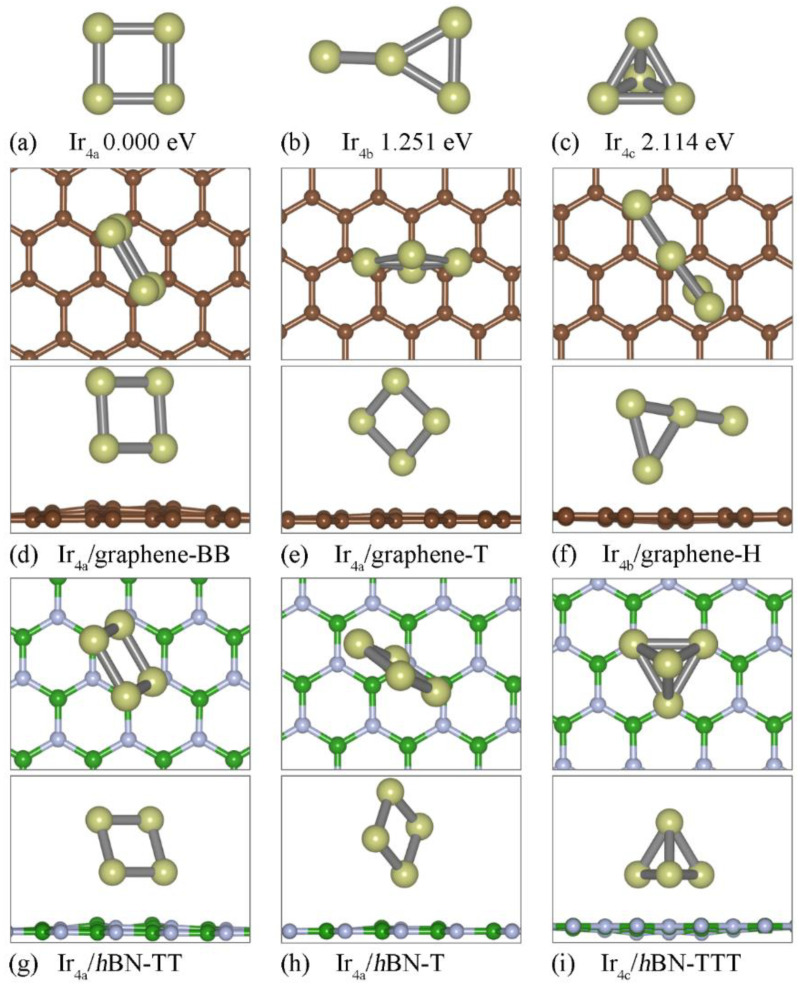
Atomic structures of the (**a**) Ir_4a_ (square planar model), (**b**) Ir_4b_ (a triangle configuration adding the fourth atom attached with the vertex of the triangle) and (**c**) Ir_4c_ (tetrahedral model) clusters. Top and side view of the three relative stable atomic structures of the Ir_4_ cluster adsorbed on the graphene (**d**–**f**) and *h*BN (**g**–**i**) substrate, respectively.

Figure 2 shows three kinds of relative stable Ir_4_ configurations for the free−standing clusters, and those absorbed onto the graphene and *h*BN substrates, respectively. For the free−standing Ir_4_ clusters, the square planar (Ir_4a_, Figure 2a) is the most favorable configuration and has an average bond length of 2.338 Å. The most stable configuration and structure parameters are consistent with those in the previous report [22]. Unlike the Ir_3_ cluster, the most stable configurations of Ir_4a_ remain unchanged on both the graphene and *h*BN substrate, along with a slight band angle deformation when they are adsorbed onto *h*BN. Figure 2e, h indicate that the metastable Ir_4_ on either the graphene or *h*BN is still a square planar configuration. The difference is that that are two Ir atoms bonded with the substrate in the most stable configuration, but there is only one in the metastable structure. Therefore, ΔE decreases from 1.251 eV (free−standing Ir_4b_) to 0.411 (Ir_4a_/*h*BN-T) and 0.093 eV (Ir_4a_/graphene-T), respectively. Similar to Ir_3b_/*h*BN-TT, for Ir_4a_/*h*BN-TT (Figure 2g), the Ir_4a_ cluster plane has a tilt angle of 75.1° with the *h*BN sheet because of the strong Ir-N interactions. As shown in Table 1, Ir_4_/*h*BN has a higher E_b_ compared with Ir_4_/graphene, which indicates a stronger interaction between the cluster and the substrate. Note that, because the configuration of the Ir_n_ cluster in Ir_n_-2D may be different, a higher E_b_ indicates a stronger interaction between the cluster and the substrate and does not guarantee a higher stability. For example, as listed in Table 1, Ir_4c_/*h*BN-TTT (Figure 2i) has a larger E_b_, along with a higher total energy.

**Figure 3:**
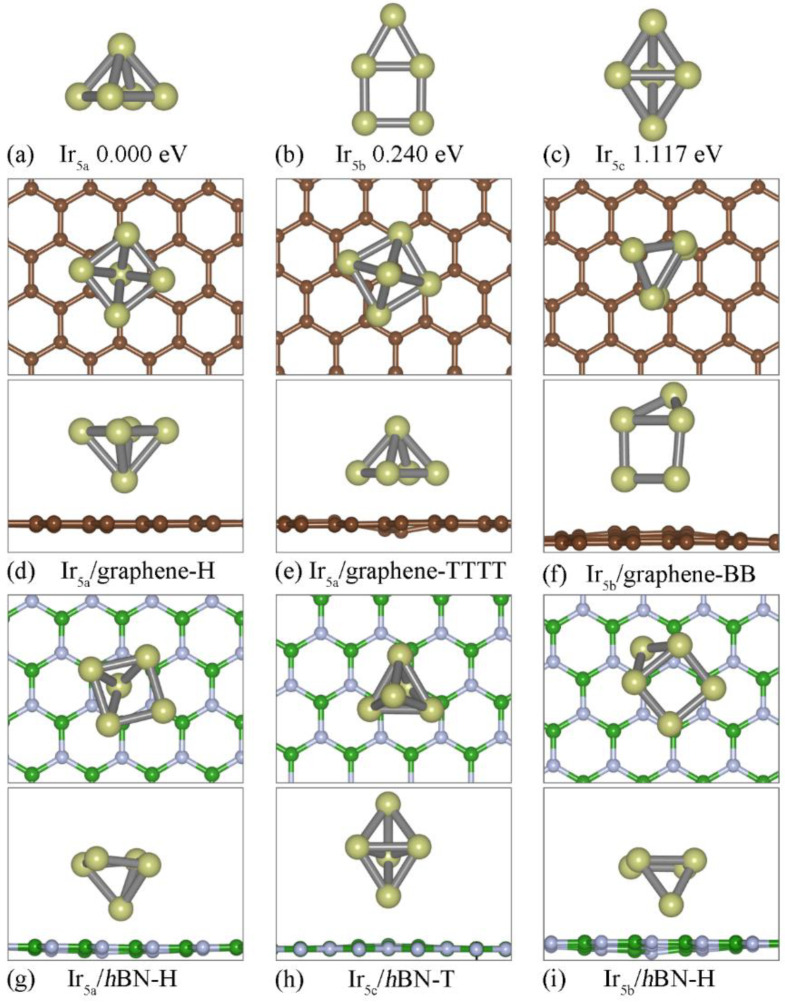
Atomic structures of the three relative stable atomic structures of the Ir_5_ clusters for the free−standing (**a**–**c**), graphene ((**d**–**f**), top and side view) and *h*BN ((**g**–**i**), top and side view) substrate, respectively.

The free−standing Ir_5_ isomers are listed in Figure 3a–c, in which Ir_5a_ (square pyramid model) is the most stable configuration, and the metastable ones are Ir_5b_ (square adding a co-plane triangle model, with an ΔE of 0.24 eV) and Ir_5c_ (triangular bipyramid model, with an ΔE of 1.117 eV), respectively. When it is absorbed on the 2D substrates, the square pyramid Ir_5a_ is still the most stable. A previous theoretical calculation also suggested that the square pyramid model of Ir_5_ is more stable for both free−standing clusters [22,33,41] or on the monolayer graphene [43]. As shown in Figure 3d,g, the bottommost Ir atom of the cluster is located at the H site of the graphene (*h*BN) with a *d* of 1.989 (1.736) Å. Ir_5c_/*h*BN-T is metastable owing to its higher *E*_b_ (6.567 eV), which is larger than that of Ir_5b_/*h*BN-H (5.622 eV).

**Figure 4:**
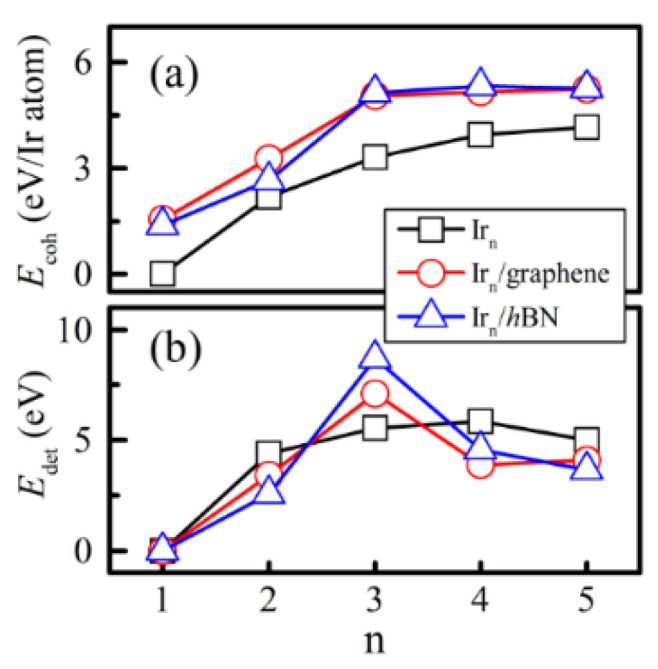
*E*_coh_ (**a**) and *E*_def_ (**b**) of the most stable structure for the Ir_n_, Ir_n_/graphene (*h*BN) clusters (n = 1–5).

We employ *E*_coh_ and *E*_det_ to further discuss the favorable Ir_n_ clusters on the substrates. As shown in Figure 4a, *E*_coh_ increases with the number of cluster atoms (n) for both the free−standing Ir_n_ [33] and Ir_n_/2D hybrid systems. Compared with the free−standing Ir_n_ cluster, Ir_n_/2D possesses a higher *E*_coh_, suggesting that the substrate effect can make the Ir_n_ cluster energetically stable. The *E*_coh_ of Ir_5_ on graphene (*h*BN) is 5.19 (5.34) eV/atom, which is comparable with that from Ghazi’s works [43,44]. As plotted in Figure 4b, for free−standing clusters, that the maximum of *E*_det_ belongs to the Ir_4_ configuration indicates that Ir_4_ is the favorite Ir_n_ cluster. After being absorbed on the substrates (either graphene or *h*BN), Ir_3_/2D turns out to be the most stable configuration due to it having the largest *E*_det_.

### 3.2. Magnetic Properties

We next discuss the magnetic properties of Ir_n_ clusters on graphene and *h*BN. By setting different spin directions in Ir_n_, we can determine what the magnetic ground state (ferromagnetic or anti-ferromagnetic) is. For all the stable configurations, including the free−standing Ir_n_ and Ir_n_ on the substrates, the ferromagnetic ground state is more energetically favorable, as shown in Supplementary Table S1. The total magnetic moments of the free−standing Ir_3a_ and Ir_3b_ are 0.947 μ_B_ and 2.654 μ_B_, respectively. The total magnetic moments of Ir_4a_, Ir_4b_ and Ir_4c_ are 6.509 μ_B_, 3.583 μ_B_ and 0 μ_B_, respectively. These results are consistent with the previous calculations [22,33]. Moreover, the total magnetic moments of Ir_5a_, Ir_5b_ and Ir_5c_ change to be 5.574 μ_B_, 7.641 μ_B_ and 7.562 μ_B_, respectively. As listed in Table 1, except for Ir_3a_/graphene-HH and Ir_3b_/*h*BN-TT, the magnetic moments of the Ir_n_ clusters on the 2D substrates decrease more or less. Compared with the free−standing Ir_n_ cluster, the magnetic moments of the most stable Ir_n_ clusters adsorbed onto the substrates were reduced to 53% (Ir_3b_/graphene-H) and 23.6% (Ir_4a_/*h*BN-TT), respectively. Furthermore, for metastable Ir_4b_/graphene-H, the magnetic moment of the Ir_4b_ cluster is only 0.548 μ_B_ (84.7% reduction). The variation of the magnetic moment caused by the substrate effect or adsorption site can be understood by the charge transfer between the cluster and the 2D sheet.

Table 1 lists the charge transfer (*d*) between Ir_n_ and the substrates from the Bader analysis [45]. Interface bonds may form between the Ir_n_ cluster and the substrate due to the electron transferring. Firstly, the magnitude of *d* on graphene is generally larger than that on *h*BN and relies on the absorption site. Secondly, for the considered Ir_n_/graphene, electrons transfer from the Ir_n_ cluster to the graphene sheet, corresponding to a negative *d*. On the contrary, the transfer direction depends on the bonded atom number when the clusters are absorbed onto the *h*BN substrate. Specifically, if one Ir atom is attached to the substrate, such as in Ir_3b_/*h*BN-H (as shown in Figure 1), the charges transfer from *h*BN to Ir_n_ (*d* > 0). If two or more atoms are attached to the substrate, such as in Ir_3b_/*h*BN-TT, the transfer direction is reversed (*d* < 0).

**Figure 5:**
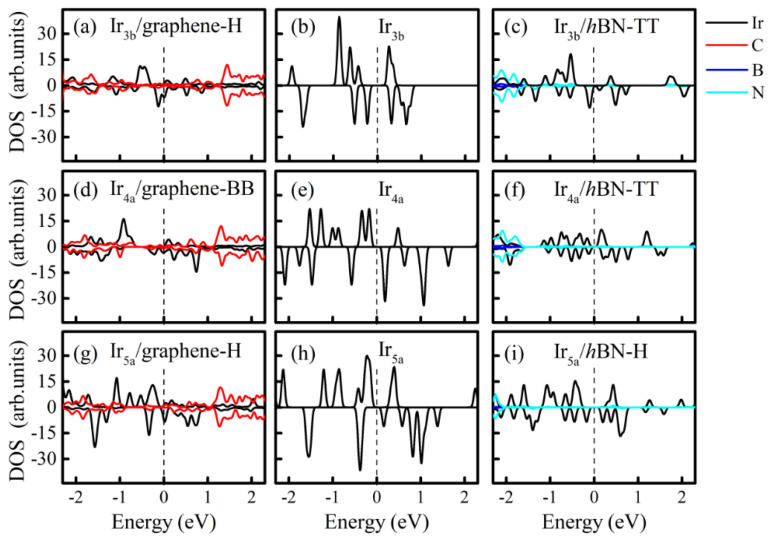
DOS for free standing Ir_n_ clusters (**b**,**e**,**h**) and Ir_n_/graphene (**a**,**d**,**g**) and Ir_n_/*h*BN (**c**,**f**,**i**) hybrid systems. Contributions of Ir, C, B and N atoms are highlighted in black, red, blue and cyan, respectively.

**Figure 6:**
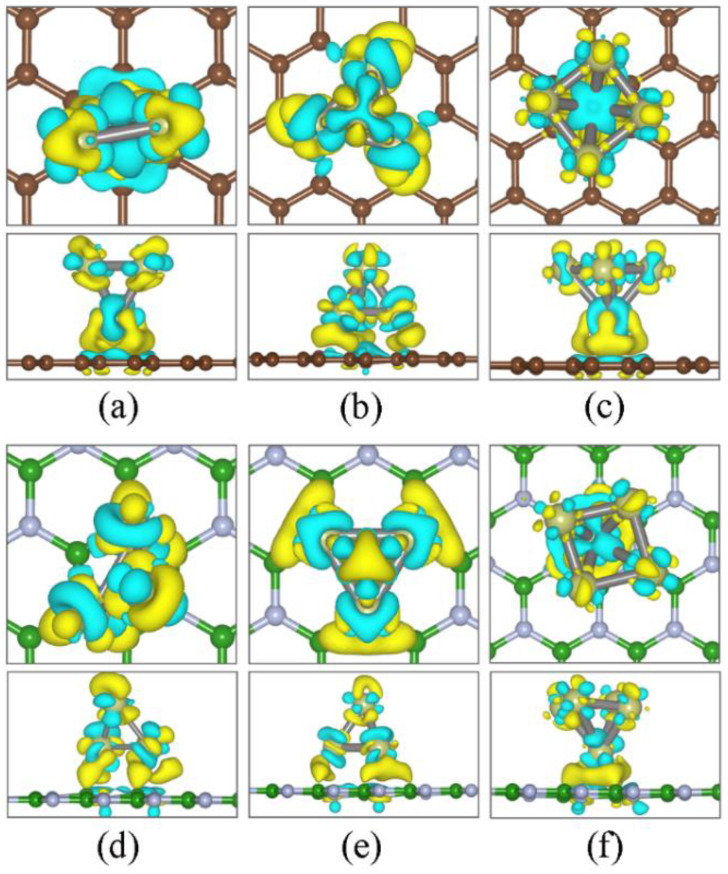
CDD of the most stable structure of Ir_3_ to Ir_5_ adsorbed on graphene (**a**–**c**) and *h*BN (**d**–**f**). Every figure shows the top and side views of CDD, respectively. Yellow and blue isosurfaces represent positive and negative charge densities. The isosurface is set at 0.005 e Å^−3^.

The density of states (DOS) of the most stable free−standing Ir_n_ and Ir_n_/2D hybrid systems is shown in Figure 5. Generally speaking, the DOS of the Ir_n_ cluster is perturbed due to the substrate effect. The substrate effect can be divided into two parts: energy level repulsion and charge transfer. From the DFT calculations, the projected DOS indicated that *d*_xz_ states are induced from the energy level repulsion between the *d*_z_^2^ states of Ir_n_ and the *p*_z_ of C (N) atoms. Meanwhile, the energy level repulsion shifts the states and increases or decreases the magnetic moments of Ir_n_. Taking Ir_3_ as an example, the magnetic moment of Ir_3b_ (2.654 mB) is increased to 2.687 μ_B_ on *h*BN but decreased to 1.408 μ_B_ on graphene. Similar changes can also be found for the Ir_4_ and Ir_5_ clusters (see Table 1). The charge transfer can be quantitatively characterized by the Bader analysis (Table 1) and qualitatively characterized by the Charge Density Difference (CDD). The CDD in Figure 6 demonstrates that charge redistribution takes place at both the interface region and the Ir_n_ cluster. Note that, differently from other Ir_n_/2D hybrid systems, the electrons in the Ir_5a_/*h*BN-H structure transfer from *h*BN to Ir atoms, as listed in Table 1. Accordingly, positive charge density dominates the interface region in Figure 6f.

**Figure 7:**
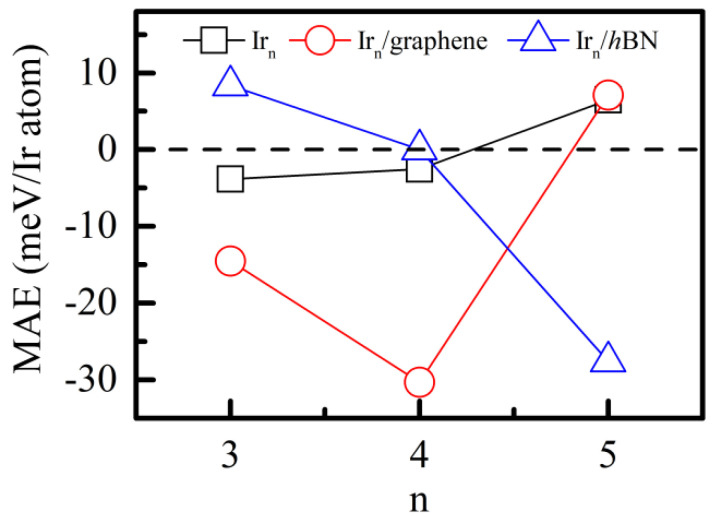
**The** MAE of the most stable free−standing Ir_n_ and Ir_n_/2D systems (n = 3–5), respectively. The zero MAE is labeled by a dashed line.

Finally, we discuss the substrate effect on the magnetic anisotropy energy (MAE) of the Ir_n_ clusters. MAE is defined as the energy difference between different easy axes (parallel (//) and perpendicular (⊥) to the 2D substrate) per Ir atom, i.e., MAE (in meV/Ir atom) = E_//_ − E_⊥_. The MAE values of the free−standing Ir_3b_, Ir_4a_ and Ir_5a_ are 11.57, 10.05 and 32.43 meV, respectively, which are consistent with previous theoretical calculations [22]. However, owing to the slight structural difference, the MAE of the free−standing Ir_4b_ (9.32 meV) is lower than that yielded from Ge’s calculation (40.26 meV) [41]. Figure 7 plots the MAE of the free−standing Ir_n_ clusters, Ir_n_/graphene and Ir_n_/*h*BN. Clearly, with the increase in n (from 3 to 5), the Ir_n_ clusters experience an easy-axis direction change. More importantly, under the influence of the substrate effect, the MAE is enlarged by about 4 times for the Ir_3b_/graphene and by 12 times for the Ir_4a_/graphene. 

**Figure 8:**
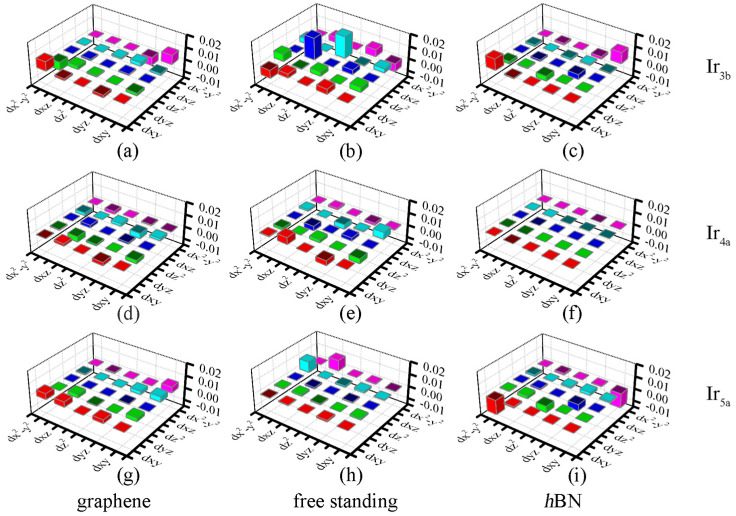
The angular momentum operator matrix of the *d* orbital of the most stable structure: (**a**) Ir_3b_/graphene-H, (**b**) free−standing Ir_3b_, (**c**) Ir_3b_/*h*BN-TT, (**d**) Ir_4a_/graphene-BB, (**e**) free−standing Ir_4a_, (**f**) Ir_4a_/*h*BN-TT, (**g**) Ir_5a_/graphene-H, (**h**) free−standing Ir_5a_ and (**i**) Ir_5a_/*h*BN-H. The units of the coordinates are eV.

MAE can be understood with the help of the second-order perturbation approach [46], which is defined as:

(4) $$\mathrm{MAE}=\xi^{2}\underset{U,O}{\sum }\frac{{\left|<O\left|l_{z}\right|U>\right|}^{2}-{\left|<O\left|l_{x}\right|U>\right|}^{2}}{E_{U}-E_{O}}$$

where ξ is the SOC constant, O (U) stands for the occupied (unoccupied) states, *E*_O_ (*E*_U_) stands for the corresponding energy eigenvalues and *l_z_* (*l_x_*) is the orbital angular momentum operator. As described by Equation (4), the coupling spin orbital matrix element difference, i.e., |<O|*l_z_*|U>|^2^ − |<O|*l_x_*|U>|^2^, contributes to the value of MAE, including different coupling orbitals and various coupling factors. Figure 8 shows the *d* orbital-resolved MAE of the free−standing Ir_n_ cluster and Ir_n_/graphene (Ir_n_/*h*BN). As shown in Figure 8b, the main contribution of the free−standing Ir_3b_ cluster to the MAE comes from the matrix element difference between the *d*_xz_ and *d*_z_^2^ orbitals. However, Figure 8a,c indicate that the main contributions on the substrate changed to *d_x_*_y_ and *d*_x_^2^_−y_^2^ orbitals for both the *h*BN and graphene, owing to the *d*_z_^2^-to-*d*_xy_ orbitals transition, as discussed above. For Ir_4a_, Figure 8e (free−standing) and Figure 8f (Ir_4a_/*h*BN) suggested that the interaction between *d*_xz_ and *d*_xy_ is the main contribution for MAE, which results in a smaller MAE. In Ir_4a_/graphene, we found that the *d* orbital is closer to the Fermi level, resulting in a higher MAE. Finally, the interactions between *d*_x_^2^_−y_^2^ and *d_xz_* (*d_xy_*) determine the MAEs of the free−standing (on substrates) Ir_5a_ clusters. The positive (negative) contributions given by the matrix element difference between different orbitals and coupling factors result in different MAE values, as discussed in reference [46].

## 4. Conclusions

In conclusion, the structural and magnetic properties of Ir_n_ (n = 3–5) clusters adsorbed on 2D substrates (graphene and *h*BN) were systematically investigated using the DFT method. The calculated results show that, after the structure relaxation, the stability order of Ir_n_ may change on 2D substrates. The detachment energies suggest that, for the free−standing Ir_n_, the most favorite cluster is the one of n = 3. After being absorbed on 2D substrates, the most stable cluster changes to n = 4. The magnetic moments of Ir_n_ generally decrease owing to the charge transfer between the Ir_n_ and the substrates, which depends on the substrate type and adsorption site. The MAE of the Ir_n_ cluster can be enlarged by 12 times for Ir_4a_/graphene, which is understood with the help of the second-order perturbation approach.

## Figures and Tables

**Table 1:** The energy difference between the most stable configuration and its isomer (ΔE, eV), the binding energy (*E*_b_, eV), the minimum distance between the cluster and the substrate (*d*, Å), the magnetic moment of the clusters (μ, μ_B_) and the hybrid system (μ_tot_, μ_B_) and the transferred charge from the cluster to the 2D substrate (δ, *e*). The negative δ represents the electrons transferring from the cluster to the 2D substrate.

	ΔE	*E*_b_	*d*	μ	μ_tot_	δ
Ir_3b_/graphene-H	0	5.352	1.771	1.408	1.287	−0.163
Ir_3b_/graphene-BH	0.159	5.192	2.007	2.385	2.517	−0.221
Ir_3a_/graphene-HH	0.278	4.950	1.714	1.017	1.083	−0.269
Ir_3b_/*h*BN-TT	0	5.583	2.248	2.687	2.713	−0.0305
Ir_3b_/*h*BN-H	0.268	5.315	1.957	2.644	2.702	0.101
Ir_3a_/*h*BN-T	0.691	4.768	2.253	0.822	0.829	0.107
Ir_4a_/graphene-BB	0	4.818	2.662	4.929	4.973	−0.245
Ir_4a_/graphene-T	0.093	4.725	2.080	4.943	4.941	−0.104
Ir_4b_/graphene-H	0.462	5.607	1.895	0.548	0.259	−0.037
Ir_4a_/*h*BN-TT	0	5.546	2.363	1.535	1.519	−0.023
Ir_4a_/*h*BN-T	0.411	5.135	2.241	2.877	2.900	0.010
Ir_4c_/*h*BN-TTT	0.938	6.722	2.075	0	0	−0.133
Ir_5a_/graphene-H	0	5.434	1.989	4.516	4.539	−0.173
Ir_5a_/graphene-TTTT	0.586	4.847	2.374	2.181	2.196	−0.468
Ir_5b_/graphene-BB	0.645	5.029	2.595	2.452	2.452	−0.170
Ir_5a_/*h*BN-H	0	5.554	1.736	3.861	3.844	0.093
Ir_5c_/*h*BN-T	0.103	6.567	2.298	2.377	2.358	0
Ir_5b_/*h*BN-H	0.172	5.622	1.742	3.940	3.933	0.067

## Data Availability

Not applicable.

## Supplementary Materials

The following supporting information can be downloaded at: https://www.mdpi.com/article/10.3390/nano12142436/s1, Figure S1: Three optimized configurations of Ir_3_ on Germanene; Table S1: The relative energies (in meV) of ferromagnetic (FM) and anti-ferromagnetic (AFM) states for the free−standing Ir_n_ and Ir_n_ on graphene and *h*BN substrates. We set the energy of FM (*E*_FM_) state as zero.
