# Supplementary material for: First-Principles Study of Irn (n = 3–5) Clusters Adsorbed on Graphene and Hexagonal Boron Nitride: Structural and Magnetic Properties
Source: Nanomaterials (Basel). 2022 Jul 16;12(14):2436. doi: 10.3390/nano12142436 (PMC9317977; doi:10.3390/nano12142436)
Supplement: Supplementary file 1 [file nanomaterials-12-02436-s001.zip › nanomaterials-1807530-supplementary.pdf]

Supplementary Materials

First-principles study of Ir_n (n = 3-5) clusters adsorbed on graphene and hexagonal boron nitride: structural and magnetic properties

Mei Ge [1,2], Leiting Chu [1,2], Miaomiao Guo [1,2], Yan Su [3] and Junfeng Zhang [1,2*]

[1] School of Physics and Information Engineering, Shanxi Normal University, Taiyuan, 030031, China; gemei@sxnu.edu.cn (M. G.); chult123@163.com (L. C.); guomm95@163.com (M. G.)

[2] Key Laboratory of Spectral Measurement and Analysis of Shanxi Province, Shanxi Normal University, Taiyuan, 030031, China

[3] Key Laboratory of Materials Modification by Laser, Electron, and Ion Beams (Dalian University of Technology), Ministry of Education, Dalian, 116024, China; su.yan@dlut.edu.cn

* Correspondence: zhangjf@sxnu.edu.cn; Tel.: +86-13935705526

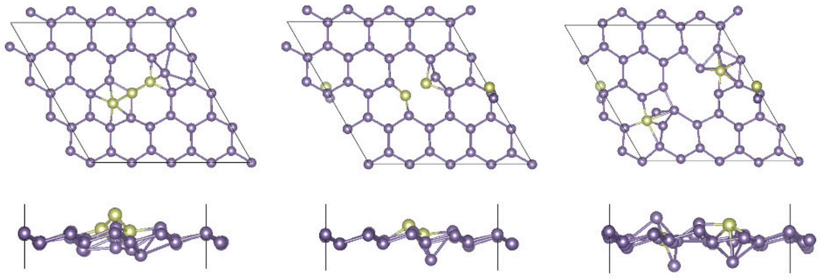

Figure S1 Three optimized configurations of Ir_3 on Germanene.

Table S1 The relative energies (in meV) of ferromagnetic (FM) and anti-ferromagnetic (AFM) states for the free–standing Ir_n and Ir_n on graphene and *h*BN substrates. We set the energy of FM (E_{FM}) state as zero.

n	Free standing		On graphene		On *h*BN	
	E_{FM}	E_{AFM}	E_{FM}	E_{AFM}	E_{FM}	E_{AFM}
3	0	0.0002	0	0.0114	0	0.0198
4	0	511.916	0	16.7884	0	255.587
5	0	0.0659	0	0.0182	0	0.0022
